# Reducing the Healthcare-Associated Infections in a Rehabilitation Hospital under the Guidance of Lean Six Sigma and DMAIC

**DOI:** 10.3390/healthcare9121667

**Published:** 2021-12-01

**Authors:** Giuseppe Cesarelli, Rita Petrelli, Carlo Ricciardi, Giovanni D’Addio, Orjela Monce, Maria Ruccia, Mario Cesarelli

**Affiliations:** 1 Department of Chemical, Materials and Production Engineering, University of Naples Federico II, Piazzale Tecchio 80, 80125 Naples, Italy; 2 Department of Electrical Engineering and Information Technology, University of Naples Federico II, Via Claudio 21, 80125 Naples, Italy; carloricciardi.93@gmail.com (C.R.); cesarell@unina.it (M.C.); 3 Scientific Clinical Institute Maugeri sb SPA, Via Generale Bellomo, 73/75, 70124 Bari, Italy; rita.petrelli@icsmaugeri.it (R.P.); gianni.daddio@icsmaugeri.it (G.D.); orjela.monce@icsmaugeri.it (O.M.); maria.ruccia@icsmaugeri.it (M.R.)

**Keywords:** lean thinking, six sigma, infections, rehabilitation hospital, public health

## Abstract

The reduction of healthcare-associated infections (HAIs) is one of the most important issues in the healthcare context for every type of hospital. In three operational units of the Scientific Clinical Institutes Maugeri SpA SB, a rehabilitation hospital in Cassano delle Murge (Italy), some corrective measures were introduced in 2017 to reduce the occurrence of HAIs. Lean Six Sigma was used together with the Define, Measure, Analyze, Improve, Control (DMAIC) roadmap to analyze both the impact of such measures on HAIs and the length of hospital stay (LOS) in the Rehabilitative Cardiology, Rehabilitative Neurology, Functional Recovery and Rehabilitation units in the Medical Center for Intensive Rehabilitation. The data of 2415 patients were analyzed, considering the phases both before and after the introduction of the measures. The hospital experienced a LOS reduction in both patients with and without HAIs; in particular, Cardiology had the greatest reduction for patients with infections (−7 days). The overall decrease in HAIs in the hospital was 3.44%, going from 169 to 121 cases of infections. The noteworthy decrease in LOS implies an increase in admissions and in the turnover indicator of the hospital, which has a positive impact on the hospital management as well as on costs.

## 1. Introduction

The term “Healthcare-acquired infections” (HAIs)—equivalently, “Healthcare associated infections”—stands for a class of infective diseases that are not present or incubated at the time of admission to a hospital, as well as infections incubated following discharge, but referred to the incubation time at the shelter [1,2]. On the other hand, HAIs do not involve patients alone but also the hospital staff during everyday patient care [3]. Current available literature has reported six types of infections, such as catheter-associated urinary tract infections (CAUTI) [4], surgical site infections, central line-associated bloodstream infections (CLABSI) [4], hospital-acquired pneumonia (HAP), ventilator-associated pneumonia, and *Clostridium difficile* [5,6] infections, which constitute more than the 80% of overall hospital infections [7]. The combination of different factors, e.g., a contaminated hospital environment, healthcare workers who act as a transmission vehicle, as well as the presence of different types of pathogens, may facilitate the transmission and persistence of multi-resistant organisms. Indeed, among identified pathogens in intensive care units, about 70% are resistant to at least one antibiotic [1].

For this reason, HAIs constitute a major public health problem and an economic burden in hospital care due to their significant impact on the subsequent increase in the length of hospitalization, morbidity, and mortality amongst hospitalized patients [8,9]. The risk of acquiring HAIs is rising. In fact, Rosenthal et al. have recently estimated that 5% of hospitalized patients contract an infection during hospitalization, with a variable point prevalence between 7% and 9% of the hospitalized patients [10].

For the last few decades, the management of HAIs has played a central role to reduce the rate of infections. The need for tracking, security measures, and timely focused prevention actions has arisen due to growing rates of antimicrobial resistance [11,12,13]. Several scientific contributions have shown empirically that the participation in systems of active surveillance of HAIs relate, over time, with a reduction in the incidence of the phenomenon.

Surveillance systems, such as the US National Nosocomial Infections Surveillance System and the European Project Hospitals in Europe Link for Infection Control through Surveillance, aim to monitor the frequency of HAIs to grant prevention [14]. Unfortunately, in several European countries, including Italy, there is no systematic approach to the surveillance systems of HAIs, and epidemiological data focused mainly on the prevalence of infected patients’ ranges [15].

The availability of high quality, accurate systems of surveillance and monitoring, supported by integrated information systems, is essential for maintaining a high level of attention, defining dimensions and characteristics of the problem, directing interventions, monitoring progress using specific indicators, and identifying sentinel events and epidemics in a timely manner.

Data regarding Residential Rehabilitation units admitting patients coming from different acute realities, such as intensive therapies and operating theatres with conditions of disability resulting from disabling diseases which require adequate 24-h care, with stable clinical conditions are sparse. This paper aims to provide current data relating to the prevention of HAIs among patients admitted to a rehabilitation hospital. As such, we focus on hand hygiene, multidrug resistance, and *Clostridium difficile*, as well as the prevention of CLABSI, HAP, and CAUTI.

### The Methodology

Several methods have been suggested to treat and manage chronic diseases [16], innovative procedures have been introduced to reduce waste in healthcare processes [17,18] and support decision-makers in the evaluation of technologies and in the choice of appropriate therapies [19].

Lean Six Sigma (LSS) was created by combining Lean Thinking and Six Sigma methodologies: the former deals with the improvement of processes by eliminating wastes, while the latter focuses on the reduction of errors and oscillations in processes by employing statistical and managements tools [20].

The wastes recognized by Lean Thinking have been applied also in the healthcare context with an appropriate connotation [21,22]:

− Transport: movement of patients and equipment;
− Inventory: unneeded stocks and supplies;
− Motion: similar to transport though meaning movement of staff and information;
− Waiting: delays in diagnosis and treatment;
− Over production: unnecessary tests;
− Over burden: related to stresses, overwork of staff;
− Defects: it can be more dangerous in healthcare, since they are medication errors and infections.

Several studies have been conducted to investigate the benefits and the challenges of LSS in different areas [23]. Historically, its implementation has been only industry-based, as ascertained by the applications in different industrial context over years [24,25,26,27], among which one of the latest applications has been the food industry [28]; however, in more than a decade, LSS have also been employed in healthcare studies with a positive influence [29,30], as also shown by Patel et al. in their recent critical review [31]. Recently, LSS has been proposed by researchers also in combination with health technology assessment [32,33,34] for different healthcare purposes.

Trzeciak et al. analyzed patients with prolonged mechanical ventilation in a multidisciplinary intensive care unit to reduce the length of hospital stay (LOS), while Montella et al. dealt with the reduction in HAIs through LSS [30,35]. In particular, the Define, Measure, Analyze, Improve and Control (DMAIC) roadmap is the main cycle employed in Six Sigma projects as a possible problem-solving strategy. DMAIC was also used to investigate the factors influencing the risk of HAIs and introduce some corrective actions within the hospital [36]. Differently, but in the same context, Cesarelli et al. studied corrective measures to reduce sentinel germs colonization and find relationships between bacteria colonization and the number of procedures and LOS in a neonatal intensive care unit [37]. Similarly, Ferraro et al. dealt with the occurrence of HAIs in an adult intense care unit [38].

The main purpose of the study is to observe and reduce HAIs in some operational units Scientific Clinical Institutes Maugeri SpA SB, a rehabilitation hospital in Cassano delle Murge (located in the Southern Italy). While, as highlighted by literature, LSS has already proved its ability to help health policy to deal with the issue of HAIs through its DMAIC cycle, there are not applications of LSS to reduce infections in rehabilitation hospitals (to the best of the authors’ knowledge); therefore, this can be considered one of the originalities of this paper. Another strength of the current application relies on the number of patients, since more than two thousand patients were included, allowing readers to consider this study as being conducted on a large dataset. A retrospective observational study was performed on the data collected from November 2016 to June 2017 compared with the data collected in the same period of the following year. In 2017, the increase in the number of HAIs clearly underlines the need to introduce new protocols and guidelines in addition to the standard precautions, as well as to reduce the incidence of those struggles within the medical center.

## 2. Materials and Methods

The project was developed at the operational units of Rehabilitative Cardiology, Rehabilitative Neurology, Functional Recovery and Rehabilitation of the Medical Center for Intensive Rehabilitation, whose beds are respectively 40, 54 and 67.

The data of all the patients involved in the present study were collected from printed medical records and digital information system database of the Scientific Clinical Institutes Maugeri SpA SB. All the statistical analyses were conducted through SPSS (v. 25).

### 2.1. Define

A multidisciplinary team approached this study. First, a project charter (Table 1) was designed to share the knowledge of the project’s details: Critical to Quality (CTQ), question, target, in and out of scope, timeline.

The team was composed by engineers, clinicians, and nurses.

In this phase, an input process output scheme was designed to clarify which were the main parts of the process [39]:

- Input: Rehabilitation services;
- Process: Rehabilitative process;
  ○ Admission to the rehabilitation hospital;
  ○ Definition of the rehabilitation program;
  ○ Rehabilitation exercises;
  ○ Discharge.
- Output: Recovery of functionality—Home program—Gain of health.

The aim of the project was to reduce the number of HAIs in the above-mentioned operational units and the LOS of patients where possible.

### 2.2. Measure

During the Define phase, the team was defined, as was the purposes of the work, while, during the Measure phase, measurements were performed to evaluate the current process.

In this phase, the data of 1278 patients from November 2017 to June 2018 were collected while, after the implementation of the new protocol (November 2018–June 2019), information was collected from a sample of 1137 patients to evaluate the influence of the improvement of LOS and infections.

The following variables were collected for each patient:

- Gender and age;
- Presence of cardiovascular, pneumological, and neurologic diseases, diabetes;
- Presence of risk factors such as urinary catheter, vascular catheter, and decubitus;
- Operational unit of afference;
- Date of admission;
- Date of surgery;
- Date of discharge.

Statistical analyzes were carried out to estimate the mean LOS, the deviation standard, and to achieve a better characterization of the chosen CTQ.

The data were considered normally distributed due to the large sample size acquired.

### 2.3. Analyze

**Figure 1:**
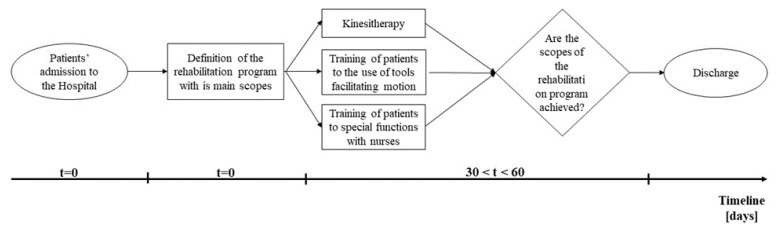
Basic stream map describing the process of hospitalization, from admission to discharge, in the rehabilitation hospital.

In this phase, the process was examined to identify each factor that could imply any issues. Firstly, a simple basic stream map (Figure 1) was drawn to visually describe the workflow of patients and evaluate which were the main issues.

The “as-is” process was described through this tool.

**Figure 2:**
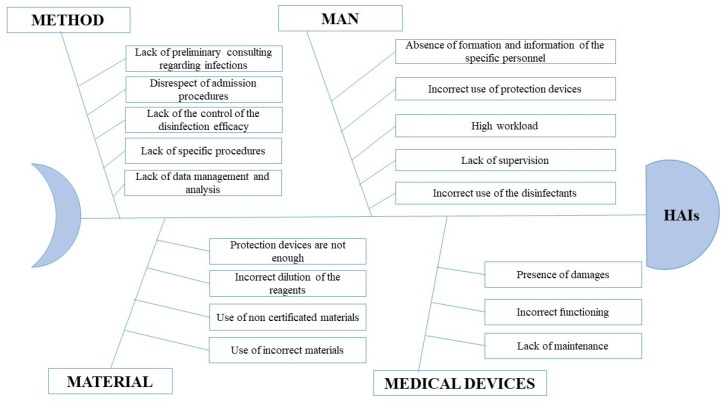
Ishikawa diagram representing the primary and the secondary causes of HAIs.

Finally, a brainstorming session with the hospital staff was conducted to discuss the causes of HAIs, and an Ishikawa diagram was drawn to represent them (Figure 2).

Four primary causes were found:

1. Method;

2. Man;

3. Material;

4. Medical Devices.

The analysis of Ishikawa (Figure 2), through the path “problem-analysis-solution”, allowed us to understand that each major cause had some secondary causes. The most relevant secondary causes were identified as the following: absence of specific formation and information of the personnel regarding the use of protection devices and disinfectants; absence of specific procedures for detecting and managing infected patients; wrong use of medical devices for disinfection.

These issues recall the ones reported by Chiarini and Filingham [21,22]: defects, waiting and over-production for patients, and over-burden for healthcare staff. Indeed, the HAIs are responsible for the prolongation of LOS (waiting), the arise of costs for unnecessary tests (over-burden), use of antibiotics (over-production), increase in the use of devices for individual protection (over-burden), a rise in mortality (defects), worsening of patients’ health status which can, eventually, result also in legal issues for the hospital (defects).

The association of some variables with infections was analyzed with a Chi square test with both *p*-values < 0.001 (Table 2).

The presence of neurological diseases, cardiovascular diseases, urinary catheter, and decubitus complications were associated to the onset of an infections, according to the statistical tests conducted in Table 2 (all *p*-values < 0.001). These results confirmed the necessity to introduce some corrective actions in the operational units of Cardiology, Neurorehabilitation, and Functional Rehabilitation.

### 2.4. Improve

The infection determines an extension of the stay itself, which could have a course up to a maximum of 180 days. In addition, these patients move to the hospital’s common environments independently, and the gyms themselves could be a place with a high risk of contagion. All these particular cases heavily affect costs (prolonged LOS, antibiotic therapies, post-exposure prophylaxis, etc.). In 2017, the Operational Instruction “Surveillance Protocol of Patients Colonized or Infected by Sentinel Microorganisms” was introduced within the Scientific Clinical Institute Maugeri. It was developed in light of the Italian Ministerial Decree of 26 February 2013 “Surveillance of Infections by CPE Bacteria”, and the Italian Legislative Decree 81/08 for safety at the workplace, which provides operational arrangements for the management of this type of patients.

The objective of this instruction was to prevent and control the direct or indirect transmission of micro-organisms, between patients and operators, through a synergic work between several parties:

− Laboratory analysis, which detects the positivity to multi-resistant germ and informs the Unit;
− The Unit, which promptly activates the appropriate isolation measures;
− The staff, on alert, which ensures the right supply of the necessary material, to achieve the proper management of the patient.

Extensive training of health professionals (doctors, nurses, social and health professionals) was also provided and extended to patients and caregivers. In particular, the focus was on the proper hand-washing procedure and on dressing and undressing procedures. A state-of-the-art sanitation system was introduced to complete the process.

Additionally, posters were affixed in the hospital to provide broad information to healthcare professionals, patients, and caregivers. Regarding the precautions to tackle HAIs, operating instructions have been disclosed regarding:

− The correct completion of the HAIs survey forms;
− Washing of hands;
− Additional precautions regarding each organism;
− The management of medicinal products and medical equipment (personal protective equipment).

Through the coordination of the Hospital Infections Committee, composed of the medical director, the medical expert, the infectious disease specialist, the nursing coordinator, a biologist and a competent doctor, the following information was monitored:

− Microbiological and antibiotic resistance data;
− The use of antibiotics for implementing guidelines;
− Cleaning and sanitizing procedures.

To complete the path, finally, a disinfection mode (99 Technologies) of contaminated surfaces and air was implemented, which allowed a reduction in the incidence of infections, thanks to the action of hydrogen peroxide and silver ions, conveyed through a dry, gas-like fog. The disinfection process consists of three distinct phases:

1. In the first phase, the solution—called 99S—is transformed into a dry fog, similar to a gas: hundreds of millions of small drops, smaller than a micron, are evenly distributed throughout the environment, thus generating a sub-micro coating;
2. In the second phase, the hydrogen peroxide acts by instantly attacking all the organic substances it encounters; the silver cations complete the bactericidal activity and cause the inactivation of viruses, bacteria, spores, fungi, and biofilms in the air and on surfaces, ensuring the prolongation of the biocidal action over time;
3. In the last phase, the free radicals generated by hydrogen peroxide rapidly transform into oxygen and no harmful residue remains in the air: it is possible to prepare the treated environment for reuse after a very short period.

The control of indoor contamination based on air and surface monitoring is carried out using environmental buffers for the detection of pathogens in hospital rooms or in places frequented by patients (gyms, halls, surgeries, etc.).

### 2.5. Control

This phase aims to validate the new procedures introduced to tackle the issue of HAIs and plan some steps to guarantee a long-term result. Our subjects were grouped according to the operational unit to understand the influence of the implemented corrective actions accordingly.

A *t*-test (uncertainty level of 0.05) was conducted to compare LOS between operated patients before and after the implementation of the corrective actions. In addition, a Chi square test was implemented to investigate the overall number of infections before and after the implementation of the corrective actions, while the frequencies related to each operational unit were compared according to the “percentage of infections”.

The team managed some actions to ensure the lung-run results that are suggested by the methodology:

− Periodical meeting to understand the need for updating the improve phase;
− Internal audits to evaluate the efficacy of the improvements;
− Using visual management tools to see updated data graphically.

## 3. Results

The comparison of LOS was performed by using a *t*-test; first, an overall evaluation was conducted and then LOS was compared according to each operational unit (Table 3).

The LOS was significantly reduced for people who did not experience an infection (*p*-value < 0.001), while infected patients experienced a non-statistically significant reduction. In regard to the operational units, infected patients of Cardiology had a decrease in LOS that was almost statistically significant (*p*-value = 0.063), while non-infected patients of Functional Rehabilitation had a statistically significant increase in LOS.

**Figure 3:**
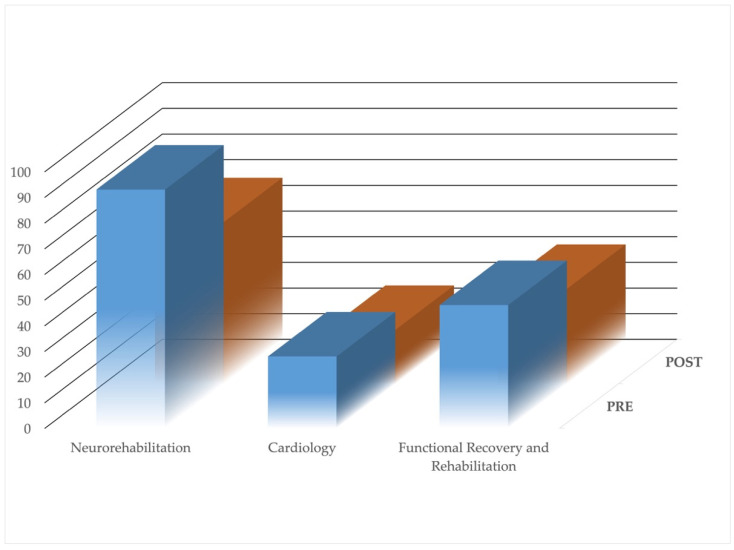
Bar plot representing the infection per each operational unit before and after the implementation of the corrective actions.

Table 4 represents the number of infections before and after the implementation of the corrective actions with a particular reference to the percentage reduction, while Figure 3 shows, visually, the same information.

By using a Chi square test, the proportion of overall infections results were statistically significant (*p*-value = 0.05). In particular, the Cardiology and Neurorehabilitation operational units experienced a reduction in infections, while the Functional Rehabilitation had a decrease in the frequencies but an increase when considering the reduction in the percentage of infection (Table 4).

Finally, the variables which could have a potential role as confounding factors were analyzed through a Chi square test or a *t*-test (Table 5).

## 4. Discussion

The proposed implementation of LSS, aimed at reducing the number of infections in a rehabilitation hospital, confirms the achievements highlighted in previous works where this methodology has already proved its feasibility and usefulness [30,36,40].

This study has shown how the new procedures, implemented to tackle HAIs, mostly corresponded to behavioral and appropriate measures to manage infected patients. Indeed, HAIs and their diffusion are strictly related to the operational modalities and to their immediate identification (i.e., swab for entering hospital services and ad hoc isolated rooms for infected patients). Moreover, HAIs’ punctual detection allows for the obtaining of useful information with which to highlight the factors which are correlated to the onset of HAIs; indeed, collecting data helps the operational units to have a historical database regarding their trend in the hospital.

Particular attention has been paid to the training of the personnel, which has allowed to achieve better management of internal resources (i.e., device for protection, correct hands washing, isolation). Personnel training aimed at correctly using the device for the disinfection of the environments has also been fundamental.

These procedures have allowed the hospital to achieve a decrease in the infections of 3.44%.

The greater improvement found for the Cardiology unit may be explained considering various factors. Firstly, the age of patients attending the departments is different and it is usually lower in the Cardiology unit, while patients’ age results higher in Neurorehabilitation and in Functional Rehabilitation units. Furthermore, the type of patients that were hospitalized in the two periods analyzed in this paper could be different (different type and percentage of devices and risk factors could affect the results); nevertheless, this particular case is intrinsic for a rehabilitative hospital which never has the same types of patients across the time. Similarly, the worse results found for the Functional Rehabilitation unit may be due to the different type of patients who usually come from an operation with an important surgical wound, which naturally requires more time to be treated.

Another explanation for the different impact of the measures among the departments may have been due to the age of the personnel. Indeed, the overall mean age of nurses and social health operators was about 45 years in the Cardiology unit and about 54 years in the Functional Rehabilitation unit. Previous studies have shown how the plasticity of the brain is reduced in older people, thus making it more difficult to learn new procedures when the personnel age increases [41].

The decrease in LOS has been a consequence of all these measures: the results showed in Table 5 provide evidence of this particular case, since the statistical tests—carried out to compare the confounding factors between the two patients’ populations—did not show statistically significant differences.

From these findings, we can deduce that the extra days which were registered for patients with HAIs—caused by the non-implementation of measures aimed at reducing HAIs in the hospital setting—resulted at an inappropriate national level for the achievement of the rehabilitation program. Obviously, the costs related to antibiotics, devices for protection, and the need for specific isolated rooms have decreased in light of the above-mentioned considerations.

While previous research was focused on reducing infections in a University Hospital [30], the novelty of this paper relies in applying the principles of LSS and DMAIC for HAIs in a rehabilitation hospital. A limitation of the study could be the absence of detailed information regarding the provenience of the patients; unfortunately, only the structure or the clinician sending patients were known, but not their clinical history. Nevertheless, this limitation is homogeneously distributed on both the pre- and the post-improvement phases, the limitation of which should not reasonably affect the study. Moreover, other confounding variables could be analyzed, but a set of five was considered sufficient to prove the benefits of the findings shown in this research.

## 5. Conclusions

As well as the previous researches conducted through LSS at the University Hospital “Federico II” [16,17,37], this work also proved that the LSS approach is able to effectively reduce costs and guide health policy-makers to elaborate new protocols or clinical pathways. The costs were analyzed for the periods of time examined in this research and, in the first period, the daily cost for a patient was 142 EUR, while, in the second period, it decreased to 14 EUR. This resulted in an overall decrease in costs: 90,000 EUR for drugs, 30,000 EUR for devices, and 328,000 EUR for personnel. Therefore, the project allowed the hospital to save 448,000 EUR.

Moreover, the noteworthy decrease in LOS implies an increase in admissions and in the turnover indicator of the hospital, which will have a positive impact on the hospital management.

A future development could be the diffusion of these improvement actions in other departments and other structures of Scientifical Clinical Institutes Maugeri, which are distributed across the whole of Italy.

## Figures and Tables

**Table 1:** Project charter.

Project title	
Reducing the infections rate in a rehabilitation hospital	
**Problem statement**	**Objective statement**
An inappropriate number of infections has been detected and needs to be reduced	Introduce corrective actions that can tackle the issue of infections
**Critical to quality**	**Target**
The CTQ is the duration of the length of hospital stay and the number of infections	Realize corrective measures in order to reduce the CTQs
**Timeline**	
Define->June 2017	
Measure->July–August 2017	
Analyze->September–October 2017	
Improve->November 2017–June 2018	
Control->November 2018–June 2019	
**In scope**	**Out of scope**
Infections Scientific Clinical Institute Maugeri: Cardiology, Neurorehabilitation and Functional Rehabilitation	1. All the other operational units 2. All the other structures

**Table 2:** The association of some variables with infections were analyzed through a Chi square test.

Variables	Categories	Infection		*p*-Value
		No	Yes	
Neurological disease	No	811	69	<0.001
	Yes	298	100	
Cardiovascular disease	No	753	140	<0.001
	Yes	356	29	
Urinary catheter	No	880	65	<0.001
	Yes	229	104	
Decubitus	No	997	139	<0.001
	Yes	112	30	

**Table 3:** Difference in LOS according to each operational unit for patients with and without infections.

Operational Unit	Infection	Before Improvement	After Improvement	*p*-Value
Overall	Yes	50.57 ± 24.27	49.12 ± 23.24	0.608
	No	40.01 ± 27.47	34.37 ± 24.72	<0.001
Cardiology	Yes	31.00 ± 14.16	24.05 ± 10.26	0.063
	No	23.54 ± 11.42	22.40 ± 10.02	0.115
Neurorehabilitation	Yes	60.91 ± 24.59	59.92 ± 23.54	0.802
	No	62.30 ± 24.43	59.28 ± 22.57	0.185
Functional rehabilitation	Yes	41.96 ± 17.05	44.95 ± 14.14	0.392
	No	36.34 ± 11.06	38.45 ± 12.74	0.014

**Table 4:** Number of infections and percentage reductions per each period according to the operational unit.

Operational Unit	Infections	Before Improvement	After Improvement	Variation (%)
Overall	Yes	169	121	−3.44
	No	1109	1016	
Cardiology	Yes	28	21	−3.60
	No	351	535	
Neurorehabilitation	Yes	93	63	−1.04
	No	260	186	
Functional Rehabilitation	Yes	48	37	+2.35
	No	498	295	

**Table 5:** Analysis of demographic and clinical factors which could influence the results.

Variables	Category	Before Improvement	After Improvement	*p*-Value
Age (mean ± dev std)	/	69.76 ± 13.59	69.92 ± 13.30	0.771
Sex	Male	681	640	0.219
	Female	587	497	
Presence of urinary catheter	Yes	260	197	0.053
	No	1008	940	
Presence of vascular catheter	Yes	333	299	0.979
	No	935	838	
Presence of decubitus	Yes	142	120	0.659
	No	1126	1017	

## Data Availability

The data presented in this study are available on request from the corresponding author. The data are not publicly available due privacy policy.

## Abbreviations

CAUTI	Catheter-associated urinary tract infections
CLABSI	Central line-associated bloodstream infections
CTQ	Critical to Quality
HAIs	Healthcare-acquired infections
HAP	Hospital-acquired Pneumonia
LOS	Length of Hospital Stay
LSS	Lean Six Sigma
